# An MPA-based optimized grey Bernoulli model for China’s petroleum consumption forecasting

**DOI:** 10.1007/s40747-022-00803-9

**Published:** 2022-07-01

**Authors:** Wen-Ze Wu, Zhiming Hu, Qin Qi, Tao Zhang

**Affiliations:** 1 School of Economics and Business Administration, Central China Normal University, Wuhan, 430079 China; 2 School of Statistics and Mathematics, Zhejiang Gongshang University, Hangzhou, 310018 China; 3 NUS Business School, National University of Singapore, 21 Lower Kent Road, Singapore, S119077 Singapore; 4 School of Economics and Management, Tongji University, Shanghai, 200092 China; 5 School of Science, Guangxi University of Science and Technology, Liuzhou, 545006 China; 6 Zhejiang College, Shanghai University of Finance and Economics, Jinhua, 321013 China

**Keywords:** Grey forecasting model, Fractional accumulated generation operator, Marine predation algorithm, Petroleum consumption

## Abstract

The remarkable prediction of petroleum consumption is of significance for energy scheduling and economic development. Considering the uncertainty and volatility of petroleum system, this paper presents a nonlinear grey Bernoulli model with combined fractional accumulated generation operator to forecast China’s petroleum consumption and terminal consumption. The newly designed model introduces a combined fractional accumulated generation operator by incorporating the traditional fractional accumulation and conformable fractional accumulation; compared to the old accumulation, the newly optimized accumulation can enhance flexible ability to excavate the development patterns of time-series. In addition, to further improve the prediction performance of the new model, marine predation algorithm is applied to determine the optimal emerging coefficients such as fractional accumulation order. Furthermore, the proposed model is verified by a numerical example of coal consumption; and this newly established model is applied to predict China’s petroleum consumption and terminal consumption. Our tests suggest that the designed ONGBM(1,1,k,c) model outperforms the other benchmark models. Finally, we predict China’s petroleum consumption in the following years with the aid of the optimized model. According to the forecasts of this paper, some suggestions are provided for policy-makers in the relevant sectors.

## Introduction

**Fig. 1:**
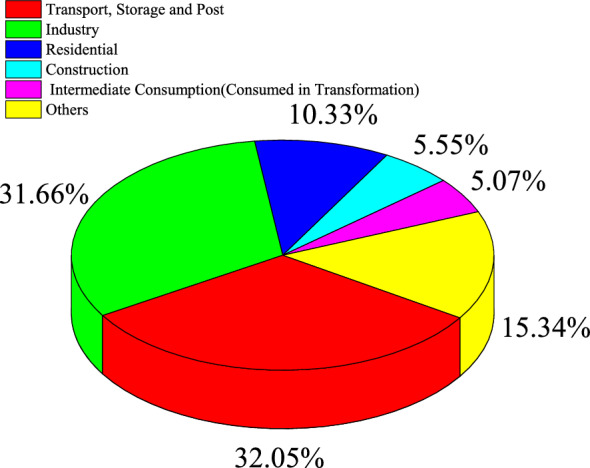
The proportion of China’s petroleum consumption by sector in 2008

**Fig. 2:**
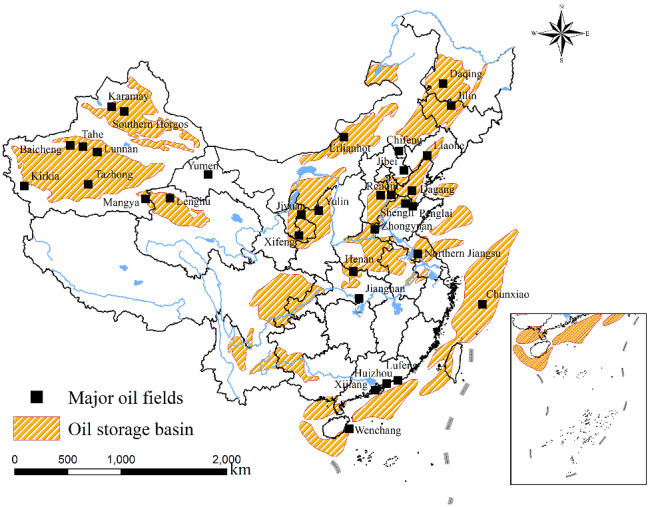
The statistical distribution of petroleum storage

In the modern industrial era, petroleum has become a very important energy resource and a strategic economic resource. The scarcity of petroleum and the sustainable development of petroleum industry directly affect the development process of the national economy and defense security [1]. On the one hand, China is on an accelerated path to industrialization and urbanization [2], and this fact undoubtedly accelerates petroleum consumption in the secondary and tertiary industries. Specifically, Fig. 1 shows the proportion of petroleum consumption in different sectors of China; it is seen that the proportion of petroleum consumption of the secondary and tertiary industries basically dominants the total volume of petroleum consumption; therefore, the development trend of petroleum consumption will increase fast. On the other hand, Fig. 2 shows that China’s petroleum resources are located in poor and remote areas, which increases the difficulty of petroleum exploration. This fact embodies that China’s petroleum demand will rely on foreign import to a large degree [3]. On the foundation of the above-mentioned background, accurately estimating China’s petroleum consumption can assist in formulating rational plans and reducing capital waste [4].

In this context, the research regarding petroleum consumption has been received more attention. Having reviewed literature, these methods can be divided into three groups: the statistical models, machine learning methods, and grey forecasting models. For example, to overcome the challenge of modeling and forecasting the oil consumption with traditional methods, Turanoglu et al*.* [5] used the artificial neural network for unfolding the oil consumption forecasting using data for population, GDP, and import and export of Turkey in the period of 1965–2010. Dritsaki et al*.* [6] modeled and forecasted oil consumption in Greece using Box–Jenkins methodology during 960–2020, and their results showed a downturn in oil consumption for the following years. Yuan et al. [7]*.* used grey model with rolling mechanism to predict global petroleum consumption. Considering the nonlinear trend in petroleum consumption. More forecasting techniques for petroleum consumption can be seen in Table 1.

**Table 1:** Recent forecasting techniques for petroleum consumption

Category	Author(s)	Model designation	Case
Statistical models	Azadeh et al*.* [8]	Fuzzy regression + *ANOVA*	Oil consumption in Canada, United States, Japan and Australia from 1990 to 2005
	Dritsaki et al*.* [6]	*ARIMA*	Oil consumption in Greece during 1960–2020
	Alkhathlan and Javid [9]	Structural time-series technique	Total oil consumption of Saudi Arabia over the period from 1971 to 2013
Machine learning methods	Al-Fattah and Aramco [10]	Artificial intelligence *GANNATS* model	Crude oil demand for Saudi Arabia and China
	Turanoglu et al*.* [5]	*ANN*s	Oil consumption in Turkey from 1965 to 2010
	Huang et al*.* [11]	Four neutral network methods	Oil consumption demand of China
Grey forecasting models	Yao and Wang [12]	*LSTM* network + *GM (1,1)*	The US West Texas Intermediate (WTI) crude oil price from January 2, 1986, to January 31, 2020
	Lu and Tsai [13]	*EWMA* + *REGM(1,1)*	Annual petroleum demand in Taiwan
	Yang et al*.* [14]	*GGNN*	China's oil consumption
	Current study	*ONGBM(1,1,k,c)*	China’s petroleum consumption and terminal consumption from 2001 to 2018

By reference with Ref. [15], there is little variation in the original data, and precise forecasts can be obtained. In addition, Ofosu-Adarkwa et al. [16] argued that it is better to focus on a time-series involving the most relevant shock when modeling a time-series sequence impacted by shocks. Taking into account data availability and volatility, this paper selects the grey modeling technique as the optimal forecasting model for accurately forecasting China’s petroleum consumption. In the past decades, grey prediction model has been widely used in various fields such as energy [17–21], environment [22], COVID-19 [23], tourism [24], automobile industry [25], and economics [26]. Due to its wide application range, a variety of derivative forms have been developed from the following aspects.

As many studies show that the traditional grey model (GM(1,1)) is suitable for processing time-series with homogeneous exponential law. To this end, Cui et al*.* [27] proposed a new model for predicting sequences with approximate non-homogeneous exponential law. Considering the influence of time-delay effect, Ma et al*.* [28] suggested time-delay polynomial grey model and applied this model in China’s natural gas consumption. Qian et al*.* [29] designed a new grey model with time power term. Wei et al*.* [30] developed a grey prediction model with polynomial term and optimized this model from the perspective of background value. Liu et al*.* [31] developed a combined grey model based on Refs. [29, 30]. Considering the nonlinear trend in the modeling sequence, Chen et al*.* [32] introduced the Bernoulli equation into GM(1,1); as a consequence, the nonlinear grey Bernoulli model (NGBM(1,1)) was proposed.

In the grey modeling procedure, the cumulative operation is applied to reduce the randomness of time-series sequence, which does achieve the purpose of accurate prediction. However, some scholars found that the prediction performance was poor when we take into account the integer-order accumulation. To address this issue, Wu et al*.* [33] introduced the fractional accumulated generation operator into the GM(1,1) model for enhancing the prediction performance. After that, Xie et al*.* [34] proposed an opposite-direction fractional grey model for forecasting China’s electricity consumption. Ma et al*.* [35] put forward a conformable fractional grey model and explored the relationship to the traditional fractional grey model.

Additionally, to estimate the model’s parameters, the trapezoid formula are usually used to approximate the integral in grey forecasting models. Zheng et al*.* [36] figured that there is an inherent error using trapezoid formula. To this end, Ma et al*.* [37] replaced trapezoid formula by Simpson formula to improve the accuracy of background value, and he and his colleagues demonstrated the feasibility and effectiveness of the newly designed model through a range of real cases. Şahin [38] used the integral mean value theorem to optimize the background value to reduce prediction errors. Subsequently, Liu et al*.* [39] further used a more complicated integral mean value theorem to enhance the prediction performance of the model.

Having reviewed the above literature, we know that the applicable scope, accumulated generation operator, and background value of the grey forecasting model still have defects. To address this issue, this paper develops a novel nonlinear grey Bernoulli model with combined fractional accumulation (abbreviated as ONGBM(1,1,k,c)). First, we introduce a combined fractional accumulated generation operator by incorporating the traditional fractional accumulation [33] and conformable fractional accumulation [35] into the optimized nonlinear grey Bernoulli model [38]. Second, the background-value coefficient is set as a variable for increasing the flexibility of the newly designed model. Third, the marine predation algorithm (MPA) [39] is applied to determine the optimal coefficients of the proposed model. Finally, to accurately predict China’s petroleum consumption in the following years, a case of coal consumption and two sets of China’s petroleum consumption are used for validating the effectiveness of the proposed model; after that, China’s petroleum consumption and terminal consumption in the next 5 years are forecasted by the newly designed model, and some suggestions are provided for policy-makers in the relevant sectors.

The rest of this paper can be organized as follows. “Optimized nonlinear grey Bernoulli model” describes the modeling procedure of the proposed model. “Numerical validation” conducts a numerical experiment for validating the effectiveness of the proposed model. “”Application in China's petroleum consumption applies the novel model to predict China’s petroleum consumption and “”Conclusion and research direction concludes.

## Optimized nonlinear grey Bernoulli model

### Combined fractional accumulated generation operator

As many studies show, the fractional accumulation generation (FAGO) has been widely used in various grey prediction models because of its flexibility and effectiveness. To further improve the performance of FAGO, this paper proposes a combined fractional accumulated generation operator (CFAGO) by incorporating the traditional fractional accumulation [33] and conformable fractional accumulation [35]. The relevant calculation formula can be seen in Theorem 1, which is helpful in establishing the proposed model in the next subsection.

#### Theorem 1.

Assume that $X^{(0)} = \left( {x^{(0)} (1),x^{(0)} (2), \ldots ,x^{(0)} (n)} \right),n \ge 4$ is nonnegative time-series sequence, and the combined fractional accumulated generation operator (CFAGO) of $X^{(0)}$ can be given by

1 $$ x^{(\alpha )} (k) = \sum\limits_{i = 1}^{k} {\sum\limits_{j = 1}^{i} {\frac{{\Gamma \left( {r + j - i} \right)x^{(0)} (k)}}{{\Gamma \left( {j - i + 1} \right)\Gamma \left( r \right)j^{[\tau ] - \tau } }}} } , $$

where $k = 1,2, \ldots ,n;\tau ,r \in (0,1]$, and its inverse form (namely, the inverse conformable fractional accumulated generation operator (ICFAGO) is obtained as

2 $$ \begin{aligned} x^{(0)} (k) &= \sum\limits_{i = 0}^{k - 1} {\left( { - 1} \right)^{i} \frac{{\Gamma \left( {r + 1} \right)\left( {k - i} \right)^{1 - \tau } }}{{\Gamma \left( {i + 1} \right)\Gamma \left( {r - i + 1} \right)}}} \\ & \quad \left( {x^{(\alpha )} (k - i) - x^{(\alpha )} (k - i - 1)} \right). \end{aligned} $$

#### Proof.

Assume that $X^{(0)} = \left( {x^{(0)} (1),x^{(0)} (2), \ldots ,x^{(0)} (n)} \right)$ is nonnegative sequence; according to [33], we can obtain the fractional accumulated generation operator of $X^{(0)}$ as $X^{(r)} = \left( {x^{(r)} (1),x^{(r)} (2), \ldots ,x^{(r)} (n)} \right)$, where

3 $$ x^{(r)} (k) = \sum\limits_{i = 1}^{k} {\frac{{\Gamma \left( {r + k - i} \right)}}{{\Gamma \left( {k - i + 1} \right)\Gamma \left( r \right)}}} x^{(0)} (i). $$

Similarly, according to [35], we get the conformable fractional accumulated generation operator (CFAGO) as

4 $$ x^{(\alpha )} (k) = \sum\limits_{i = 1}^{k} {\frac{{x^{(r)} (k)}}{{i^{[\tau ] - \tau } }}} , $$

where $\tau \in (0,1]$, that is, the CFAGO of $X^{(0)}$ is

5 $$ x^{(\alpha )} (k) = \sum\limits_{i = 1}^{k} {\sum\limits_{j = 1}^{i} {\frac{{\Gamma \left( {r + j - i} \right)x^{(0)} (k)}}{{\Gamma \left( {j - i + 1} \right)\Gamma \left( r \right)j^{[\tau ] - \tau } }}} } . $$

After an inverse calculation, we get

6 $$ x^{(r)} (k) = k^{1 - \tau } \left( {x^{(\alpha )} (k) - x^{(\alpha )} (k - 1)} \right). $$

Based on this, we easily get the inverse fractional accumulated generation operator of $X^{(r)}$ as

7 $$ x^{( - r)} (k) = \sum\limits_{i = 1}^{k} {\left( {\begin{array}{*{20}c} {k - i - r - 1} \\ {k - i} \\ \end{array} } \right)} x^{(0)} (i). $$

Then, Eq. (7) becomes

8 $$ x^{( - r)} (k) = \sum\limits_{i = 1}^{k} {\left( { - 1} \right)^{i} \frac{{\Gamma \left( {r + 1} \right)x^{(0)} (k - i)}}{{\Gamma \left( {i + 1} \right)\Gamma \left( {r - i + 1} \right)}}} . $$

Furthermore, we can obtain the predicted value of $X^{(0)}$ expressed as

9 $$ \begin{aligned} x^{(0)} (k) &= \sum\limits_{i = 0}^{k - 1} {\left( { - 1} \right)^{i} \frac{{\Gamma \left( {r + 1} \right)\left( {k - i} \right)^{1 - \tau } }}{{\Gamma \left( {i + 1} \right)\Gamma \left( {r - i + 1} \right)}}}\\ & \quad \left( {x^{(\alpha )} (k - i) - x^{(\alpha )} (k - i - 1)} \right). \end{aligned} $$

This completes the proof.

By reference with [33, 35], it is easily found that both the FAGO and CAGO are special cases of the CFAGO.

### Model establishment

Inspired by Wu et al. [38], this paper presents an optimized nonlinear grey Bernoulli model by introducing the CFAGO, and the different equation of the newly-designed ONGBM(1,1,k,c) model can be defined as

10 $$ \frac{{d\left[ {x^{(\alpha )} (t)} \right]}}{dt} + ax^{(\alpha )} (t) = \left( {bt^{2} + ct + d} \right)\left( {x^{(\alpha )} (t)} \right)^{\gamma } , $$

where $a$ is the development coefficient,$b$,$c$ and $d$ are the grey qualities, and $\gamma$ is the power index and $\gamma \ne 1$. Obviously, when $\alpha = 1$, this model is transformed into NGBM(1,1,k,c); when $\alpha = 1,b = 0$, this model is simplified as NGBM(1,1); when $\alpha = 1,b = 0,\gamma = 0$, this model is presented as NGM(1,1,k,c); and when $\alpha = 1,\gamma = 0$, this model is converted to GMP(1,1,2). Therefore, the proposed ONGBM(1,1,k,c) model has a more flexible structure and compatibility with other grey models.

### Exact solution to ONGBM(1,1,k,c)

This section gives the exact solution to the proposed model and the predicted value of the original data sequence.

First, we multiply both sides of Eq. (2) by $\left[ {x^{(\alpha )} (t)} \right]^{ - \gamma }$, and one gets

11 $$ \frac{{d\left[ {x^{(\alpha )} (t)} \right]}}{dt}\left[ {x^{(\alpha )} (t)} \right]^{ - \gamma } + a\left[ {x^{(\alpha )} (t)} \right]^{1 - \gamma } = bt^{2} + ct + d. $$

Let $\psi^{(\alpha )} (t) = \left[ {x^{(\alpha )} (t)} \right]^{1 - \gamma }$, Eq. (11) can be written as

12 $$ \frac{{d\left[ {\psi^{(\alpha )} (t)} \right]}}{dt}\frac{1}{1 - \gamma } + a\psi^{(\alpha )} (t) = bt^{2} + ct + d. $$

Furthermore, let $A = a\left( {1 - \gamma } \right)$, $B = b\left( {1 - \gamma } \right)$, $C = c\left( {1 - \gamma } \right)$, and $D = d\left( {1 - \gamma } \right)$, Eq. (12) can be then expressed as

13 $$ \frac{{d\left[ {\psi^{(\alpha )} (t)} \right]}}{dt} + A\psi^{(\alpha )} (t) = Bt^{2} + Ct + D. $$

By solving Eq. (13), we obtain the time response function of the ONGBM(1,1,k,c) model expressed as

14 $$ x^{(\alpha )} (k) = \left\{ \begin{gathered} e^{ - A(k - 1)} \left[ {\frac{B}{A}k^{2} + \frac{AC - 2B}{{A^{2} }}k + \frac{{2B - AC - + A^{2} D}}{{A^{3} }}} \right] \hfill \\ + \frac{B}{A}k^{2} + \frac{AC - 2B}{{A^{2} }}k + \frac{{2B - AC - + A^{2} D}}{{A^{3} }} \hfill \\ \end{gathered} \right\}^{{\frac{1}{1 - \gamma }}} . $$

With the help of the ICFAGO, the predicted value of the original series is given as

15 $$ \begin{aligned} x^{(0)} (k) &= \sum\limits_{i = 0}^{k - 1} {\left( { - 1} \right)^{i} \frac{{\Gamma \left( {r + 1} \right)\left( {k - i} \right)^{1 - \tau } }}{{\Gamma \left( {i + 1} \right)\Gamma \left( {r - i + 1} \right)}}}\\ & \quad \left( {x^{(\alpha )} (k - i) - x^{(\alpha )} (k - i - 1)} \right). \end{aligned} $$

### Parameter estimation

This section deduces the model’s parameters ($a$,$b$,$c$ and $d$); integrating both sides of Eq. (13) over the interval $[k - 1,k]$, one can write

16 $$ \psi^{(\alpha )} (k) - \psi^{(\alpha )} (k - 1) + A\int\limits_{k - 1}^{k} {\psi^{(\alpha )} (t)dt} = \frac{{k^{3} - (k - 1)^{3} }}{3}B + \frac{{k^{2} - (k - 1)^{2} }}{2}C + D. $$

Using the trapezoid formula, Eq. (16) can be converted as

17 $$ \psi^{(\alpha )} (k) - \psi^{(\alpha )} (k - 1) + Az^{(\alpha )} (k) = \frac{{k^{3} - (k - 1)^{3} }}{3}B + \frac{{k^{2} - (k - 1)^{2} }}{2}C + D. $$

In Eq. (17),$z^{(\alpha )} (k)$ is the background value at point $k$, and

18 $$ z^{(\alpha )} (k) = \left[ {\kappa \psi^{(\alpha )} (k) + (1 - \kappa )\psi^{(\alpha )} (k)} \right],\kappa \in [0,1]. $$

Using the least square method, we get the parameter vector $\hat{\rho } = \left( {a,b,c,d} \right)^{{\text{T}}}$ expressed as

19 $$ \hat{\rho } = \left( {U^{{\text{T}}} U} \right)^{ - 1} U^{{\text{T}}} I, $$

where

$$ U = \left[ {\begin{array}{*{20}c} { - z^{(\alpha )} (2)} & {\frac{{2^{3} - 1^{3} }}{3}} & {\frac{{2^{2} - 1^{2} }}{2}} & 1 \\ { - z^{(\alpha )} (3)} & {\frac{{3^{3} - 2^{3} }}{3}} & {\frac{{3^{2} - 2^{2} }}{2}} & 1 \\ \vdots & \vdots & \vdots & \vdots \\ { - z^{(\alpha )} (n)} & {\frac{{n^{3} - \left( {n - 1} \right)^{3} }}{3}} & {\frac{{n^{2} - \left( {n - 1} \right)^{2} }}{2}} & 1 \\ \end{array} } \right],I = \left[ {\begin{array}{*{20}c} {\psi^{(\alpha )} (2) - \psi^{(\alpha )} (1)} \\ {\psi^{(\alpha )} (3) - \psi^{(\alpha )} (2)} \\ \vdots \\ {\psi^{(\alpha )} (n) - \psi^{(\alpha )} (n - 1)} \\ \end{array} } \right]. $$

### Optimization of hyperparameters

As above-mentioned modeling steps, it is found that the four parameters are assumed to be known. By reference with [18, 40], we establish a simple optimization problem to obtain the optimal hyperparameters in the ONGBM(1,1,k,c) model. The objective function with constraints can be defined as follows:

20 $$ \begin{gathered} \mathop {\min }\limits_{\alpha ,\gamma ,\tau ,\kappa } MAPE = \frac{1}{n}\sum\limits_{k = 1}^{n} {\left| {\frac{{\hat{x}^{(\alpha )} (k) - x^{(\alpha )} (k)}}{{x^{(\alpha )} (k)}}} \right|} \times 100\% \hfill \\ s.t.\left\{ \begin{gathered} \alpha ,\tau \in (0,1],\kappa \in [0,1],\gamma \ne 1 \hfill \\ X^{(0)} = \left( {x^{(0)} (1),x^{(0)} (2), \ldots ,x^{(0)} (n)} \right) \hfill \\ x^{(\alpha )} (k) = \sum\limits_{i = 1}^{k} {\sum\limits_{j = 1}^{i} {\frac{{\Gamma \left( {r + j - i} \right)x^{(0)} (k)}}{{\Gamma \left( {j - i + 1} \right)\Gamma \left( r \right)j^{[\tau ] - \tau } }}} } \hfill \\ \hat{\rho } = \left( {U^{{\text{T}}} U} \right)^{ - 1} U^{{\text{T}}} I \hfill \\ x^{(\alpha )} (k) = \left\{ \begin{gathered} e^{ - A(k - 1)} \left[ {\frac{B}{A}k^{2} + \frac{AC - 2B}{{A^{2} }}k + \frac{{2B - AC - + A^{2} D}}{{A^{3} }}} \right] \hfill \\ + \frac{B}{A}k^{2} + \frac{AC - 2B}{{A^{2} }}k + \frac{{2B - AC - + A^{2} D}}{{A^{3} }} \hfill \\ \end{gathered} \right\}^{{\frac{1}{1 - \gamma }}} \hfill \\ x^{(0)} (k) = \sum\limits_{i = 0}^{k - 1} {\left( { - 1} \right)^{i} \frac{{\Gamma \left( {r + 1} \right)\left( {k - i} \right)^{1 - \tau } }}{{\Gamma \left( {i + 1} \right)\Gamma \left( {r - i + 1} \right)}}} \left( {x^{(\alpha )} (k - i) - x^{(\alpha )} (k - i - 1)} \right) \hfill \\ \end{gathered} \right.. \hfill \\ \end{gathered} $$

It is difficult to solve Eq. (20) because of its nonlinear characteristics. To this end, the MPA algorithm is employed to seek the optimal values of the four hyperparameters by minimizing the mean absolute percentage error (MAPE) between the fitted and original values.

MPA divides the whole system into three optimization stages based on different speed ratios, including high speed ratio, unit speed ratio, and low speed ratio. The specified algorithm is described as follows.

Initialization phase. Similar to most metaheuristic algorithms, MPA randomly initializes the prey positions within the search space to initiate the optimization process. The mathematical description is as follows:

21 $$ X_{0} = X_{\min } + rand\left( {X_{\max } - X_{\min } } \right), $$

where $X_{\max }$ and $X_{\min }$ indicate the search range, and $rand()$ is generated within the random numbers.

Optimization phase. At the beginning of the iteration, when the predator speed is faster than the prey speed, the mathematical description of the MPA optimization process based on the exploration strategy is as follows:

22 $$ \left\{ \begin{gathered} {\text{stepsize}}_{i} = R_{B} \otimes \left( {{\text{Elite}}_{i} - R_{B} \otimes {\text{Prey}}_{i} } \right) \hfill \\ {\text{Prey}}_{i} = {\text{Prey}}_{i} + P \cdot R \otimes {\text{stepsize}}_{i} \hfill \\ \end{gathered} \right., $$

where $i = 1,2, \ldots ,n$;${\text{Iter}} < \frac{1}{3}{\text{Max\_Iter}}$. In Eq. (22), ${\text{stepsize}}$ is the movement step. $R_{B}$ is the Brownian wandering random vector with normal distribution;${\text{Elite}}$ is the elite matrix constructed from the top predators.${\text{Prey}}$ is the prey matrix with the same dimension as the elite matrix.$\otimes$ is the term-by-term multiplication operator.$P$ is equal to 0.5.$R$ is a uniform random vector within [0, 1];$n$ is the population size.${\text{Iter}}$ and ${\text{Max\_Iter}}$ are the current, and maximum, numbers of iterations, respectively.

In the middle of the iteration, when the predator and the prey have the same speed, the prey is responsible for exploitation based on the Lévy wandering strategy; the predator is responsible for exploration based on the Brownian wandering strategy, and gradually shifts from the exploration strategy to the exploitation strategy. The mathematical description of exploitation and exploration is as follows:

23 $$ \left\{ \begin{gathered} {\text{stepsize}}_{i} = R_{L} \otimes \left( {{\text{Elite}}_{i} - R_{L} \otimes {\text{Prey}}_{i} } \right) \hfill \\ {\text{Prey}}_{i} = {\text{Prey}}_{i} + P \cdot R \otimes {\text{stepsize}}_{i} \hfill \\ \end{gathered} \right., $$

where $$i = 1,2, \ldots ,\frac{n}{2}$$,$$\frac{1}{3}{\text{Max\_Iter}} < {\text{Iter}} < \frac{2}{3}{\text{Max\_Iter}}$$

24 $$ \left\{ \begin{gathered} {\text{stepsize}}_{i} = R_{B} \otimes \left( {R_{B} \otimes {\text{Prey}}_{i} - {\text{Elite}}_{i} } \right) \hfill \\ {\text{Prey}}_{i} = {\text{Elite}}_{i} + P \cdot CF \otimes {\text{stepsize}}_{i} \hfill \\ \end{gathered} \right., $$

where $i = \frac{n}{2}, \ldots ,n$,$\frac{1}{3}{\text{Max\_Iter}} < {\text{Iter}} < \frac{2}{3}{\text{Max\_Iter}}$. In Eqs. (23) and (24), CF is the random vector with Lévy distribution and

25 $$ {\text{CF}} = \left( {1 - \frac{{{\text{Iter}}}}{{{\text{Max\_Iter}}}}} \right)^{{\frac{{2{\text{Iter}}}}{{{\text{Max\_Iter}}}}}} $$

is the adaptive parameter to control the movement step of the predator, and the other parameters have the same meaning as above. At the end of the iteration, when the predator speed is slower than the prey speed, the predator adopts an exploitation strategy based on Lévy wandering. The mathematical description is as follows:

26 $$ \left\{ \begin{gathered} {\text{stepsize}}_{i} = R_{L} \otimes \left( {{\text{Elite}}_{i} - R_{L} \otimes {\text{Prey}}_{i} } \right) \hfill \\ {\text{Prey}}_{i} = {\text{Elite}}_{i} + P \cdot CF \otimes {\text{stepsize}}_{i} \hfill \\ \end{gathered} \right., $$

where $$i = 1,2, \ldots ,n$$, $${\text{Iter}} > \frac{2}{3}{\text{Max\_Iter}}$$.

$FADs$ effect. Fish aggregation devices (FADs) or eddy effects usually change the foraging behavior of marine predators, and this strategy enables MPA to overcome the early convergence problem and escape from local extremes in the process of finding the optimal value. Its mathematical description is as follows:

When $$r \le FADs$$,

27 $$ {\text{Prey}}_{i} = {\text{Prey}}_{i} + CF\left[ {X_{\min } + R_{L} \otimes \left( {X_{\max } - X_{\min } } \right)} \right] \otimes U. $$

When $$r > FADs$$,

28 $$ {\text{Prey}}_{i} = \left[ {FADs(1 - r) + r} \right]\left( {{\text{Prey}}_{r1} - {\text{Prey}}_{r2} } \right), $$

where $FADs$ are the influence probabilities, taken as 0.2. $U$ is the binary vector.$r$ is the random number within [0, 1].$r1$ and $r2$ are the random indices of the prey matrix, respectively.

**Fig. 3:**
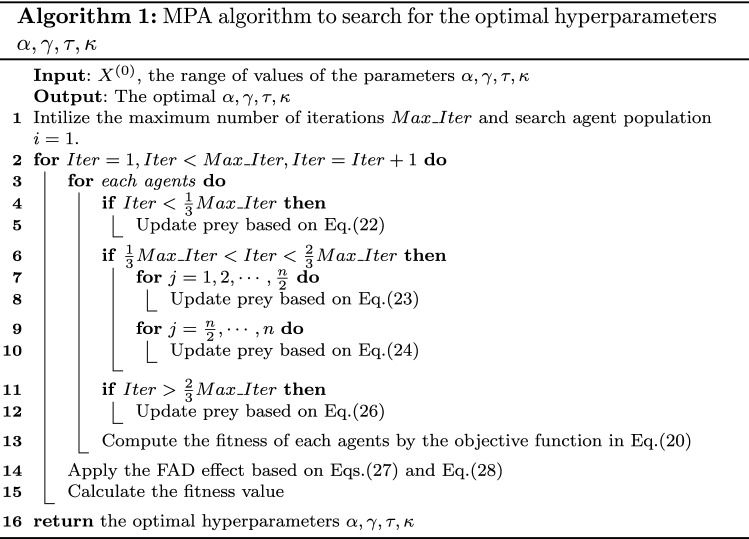
The computational steps for the MPA algorithm-based ONGBM(1,1,k,c) model

The detailed computational steps for the MPA can be seen in Fig. 3.

### Evaluation criterion

In this paper, four statistical indicators [41] are used to evaluate the effectiveness of the model, including the mean absolute percentage error (MAPE), normalized mean absolute percentage error (NMAPE), root-mean-square error (RMSE) and normalized root-mean-square error (NRMSE). And the relevant calculation formulas can be seen as follows:

29 $$ MAPE = \frac{1}{n}\sum\limits_{k = 1}^{n} {\left| {\frac{{\hat{x}^{(0)} (k) - x^{(0)} (k)}}{{x^{(0)} (k)}}} \right|} \times 100\% $$

30 $$ NMAPE = \frac{1}{n}\sum\limits_{k = 1}^{n} {\left| {\frac{{\hat{x}^{(0)} (k) - x^{(0)} (k)}}{{\sum\nolimits_{k = 1}^{n} {x^{(0)} (k)} }}} \right|} \times 100\% $$

31 $$ RMSE = \sqrt {\frac{1}{n}\sum\limits_{k = 1}^{n} {\left( {\hat{x}^{(0)} (k) - x^{(0)} (k)} \right)^{2} } } $$

32 $$ NRMSE = \frac{{\sqrt {\sum\nolimits_{k = 1}^{n} {\left( {\hat{x}^{(0)} (k) - x^{(0)} (k)} \right)^{2} } } }}{{\sqrt {\sum\nolimits_{k = 1}^{n} {\left( {x^{(0)} (k)} \right)^{2} } } }}, $$

where $x^{(0)} (k)$ and $\hat{x}^{(0)} (k)$ are the actual value and predicted value at point $k$, respectively.

## Numerical validation

This section provides an example to validate the flexibility and effectiveness of the proposed ONGBM(1,1,k,c) model. The basic grey model (GM(1,1)) [42] and optimized nonlinear grey Bernoulli model (ONGBM(1,1)) [43] are selected as the benchmark models. These computational results have computed on the software MATLAB in authors’ labs.

In this section, we consider a time-series sequence from the National Bureau of Statistics of China (http://www.stats.gov.cn/tjsj/ndsj/) that describes the changing trend of China’s coal consumption. The data from 2000 to 2016 are applied to calibrate the model, while the data from 2017 to 2019 are used for examining the model’s accuracy. From Table 2, it is concluded that the proposed model is superior over other competitive models in this case.

**Table 2:** Fitted and predicted values and errors (%) by the different grey models in China’s coal consumption

Year	Raw data	GM(1,1)		ONGBM(1,1)		ONGBM(1,1,k,c)	
		Value	APE	Value	APE	Value	APE
*Training stage*							
2000	21,232.01	21,232.01	0.00	21,232.01	0.00	21,232.01	0.00
2001	21,342.74	23,120.30	8.33	21,315.46	0.13	21,194.82	0.69
2002	22,544.05	24,606.03	9.15	23,626.75	4.80	23,224.32	3.02
2003	24,922.00	26,187.24	5.08	25,732.38	3.25	25,334.16	1.65
2004	28,749.31	27,870.06	3.06	27,777.16	3.38	27,508.17	4.32
2005	30,088.94	29,661.02	1.42	29,822.70	0.88	29,746.24	1.14
2006	32,245.20	31,567.07	2.10	31,902.62	1.06	31,792.33	1.40
2007	34,031.60	33,595.61	1.28	34,038.54	0.02	34,286.48	0.75
2008	35,498.24	35,754.50	0.72	36,246.07	2.11	36,588.54	3.07
2009	38,128.59	38,052.12	0.20	38,537.61	1.07	39,018.60	2.33
2010	42,874.55	40,497.39	5.54	40,923.73	4.55	41,448.62	3.33
2011	43,965.84	43,099.80	1.97	43,413.94	1.26	43,878.60	0.20
2012	46,678.92	45,869.44	1.73	46,017.17	1.42	46,500.56	0.38
2013	48,652.15	48,817.06	0.34	48,742.03	0.18	49,122.45	0.97
2014	51,596.95	51,954.10	0.69	51,597.05	0.00	51,680.28	0.16
2015	54,788.28	55,292.73	0.92	54,590.79	0.36	54,430.10	0.65
2016	57,125.93	58,845.90	3.01	57,731.93	1.06	57,179.84	0.09
MAPE			2.68		1.50		1.42
NMAPE			2.31		1.37		1.29
RMSE			1114.44		713.41		641.48
NRMSE			0.03		0.02		0.00
*Test stage*							
2017	59,402.17	62,627.40	5.43	61,029.40	2.74	59,865.53	0.78
2018	63,004.33	66,651.91	5.79	64,492.38	2.36	62,743.20	0.41
2019	67,268.27	70,935.03	5.45	68,130.38	1.28	65,684.80	2.35
MAPE			5.56		2.13		1.18
NMAPE			5.55		3.45		1.21
RMSE			3519.09		2242.26		964.41
NRMSE			0.06		0.04		0.00

## Application in China’s petroleum consumption

### Data collection and experimental design

The modeling data of petroleum consumption and petroleum terminal consumption from 2001 to 2018 are abstracted from the National Bureau of Statistics of China (http://www.stats.gov.cn/tjsj/ndsj/), as shown in Table 3.

**Table 3:** China’s petroleum consumption and terminal consumption from 2001 to 2018 [unit:10^4^ tons]

Year	Petroleum consumption	Terminal consumption
2001	22,888.40	20,407.10
2002	24,789.20	21,992.20
2003	27,125.80	24,062.50
2004	31,700.50	28,062.70
2005	32,547.00	29,495.60
2006	34,876.20	31,614.10
2007	36,658.70	33,857.60
2008	37,302.90	34,702.90
2009	38,384.50	35,689.90
2010	44,101.00	41,243.40
2011	45,378.50	42,727.30
2012	47,797.30	45,080.70
2013	49,970.60	47,458.80
2014	51,859.40	49,309.00
2015	55,960.20	52,945.70
2016	57,692.90	54,387.00
2017	60,395.90	56,880.00
2018	62,245.10	58,623.00

Since China’s accession to the WTO in 2001, the Chinese economy has grown rapidly, thus leading to a dramatic increase in energy demand, and petroleum is seen as the blood of industry. Therefore, accurately forecasting China’s petroleum consumption is the motivation of this paper. To this end, this paper develops a new method for estimating the trend of China’s petroleum consumption and petroleum terminal consumption. In addition to the benchmarks mentioned in “Numerical validation”, the polynomial regression (PR) [45], artificial neural network (ANN) [46], and support vector regression (SVR) [47] are also chosen for demonstrating the effectiveness of the newly designed model.

### Experimental results

**Fig. 4:**
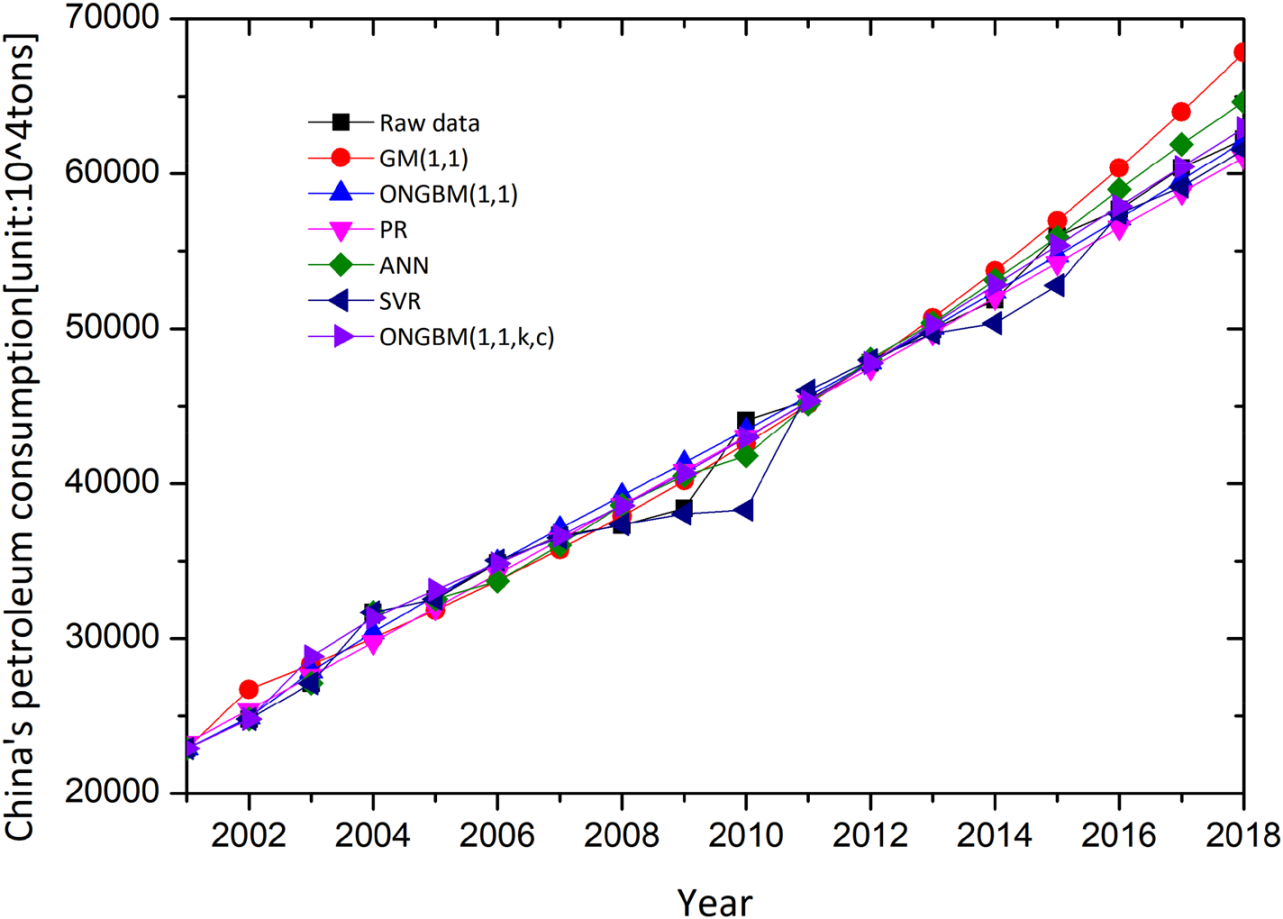
Comparison of forecasted and actual values from 2001 to 2018

**Fig. 5:**
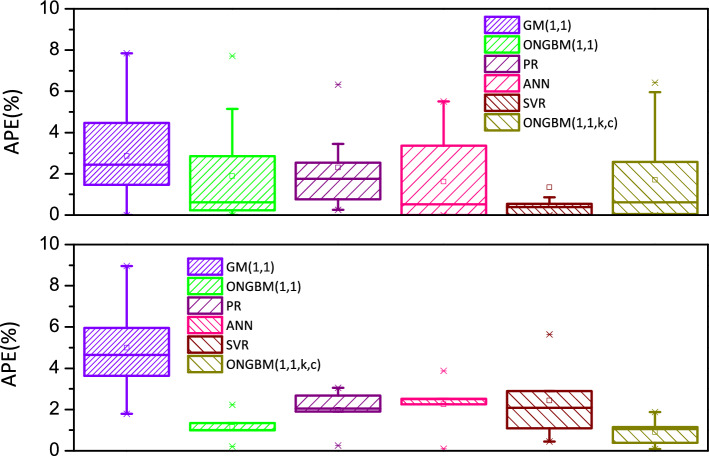
APE(%) of the six different models in China’s petroleum consumption

**Fig. 6:**
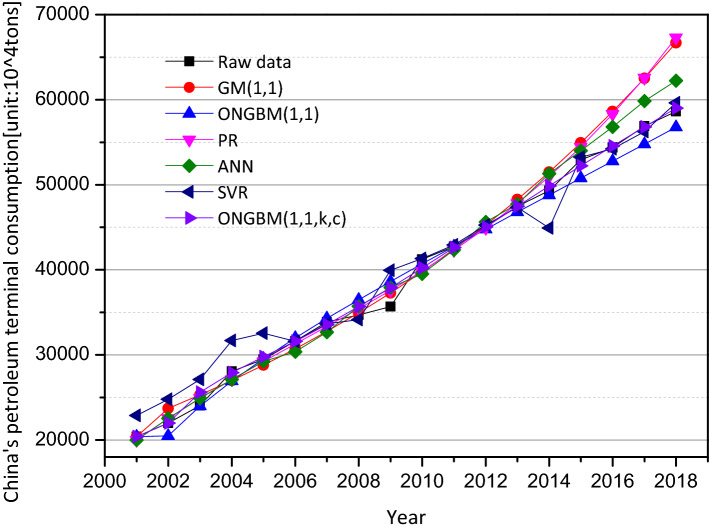
Comparison of forecasted and actual values in China’s petroleum terminal consumption

**Fig. 7:**
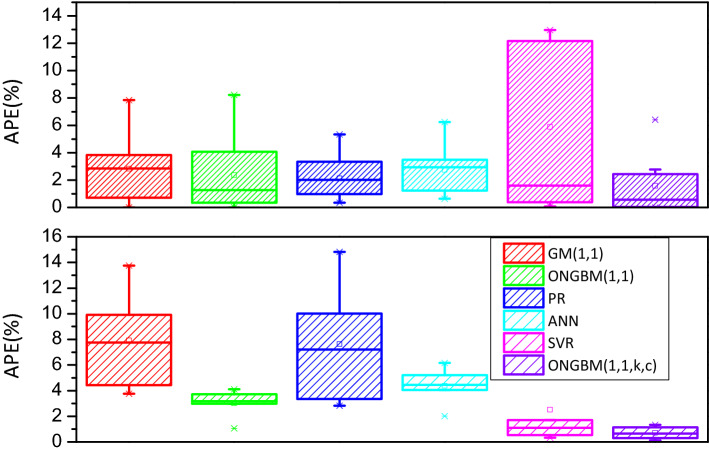
APE(%) of the six different models in China’s petroleum terminal consumption

The two datasets (annual petroleum consumption and petroleum terminal consumption) are conducted on the foundation of the mentioned competitive models, and the modeling results are listed in Tables 4, 5, 6 and 7. For visualization and intuition purposes, the forecasted values and the absolute percentage errors (APEs) generated by the six different models are graphed in Figs. 4, 5, 6 and 7. The competitive analysis will be implemented from the following angles: (1) the simulation and prediction deviations among the grey- and non-grey-based models, and (2) the effectiveness and flexibility of the proposed ONGBM(1,1,k,c) model using four calculated error-value metrics.

**Table 4:** Simulated and forecasted values of China’s petroleum consumption using different models

Year	Data	GM(1,1)		ONGBM(1,1)		PR		ANN		SVR		ONGBM(1,1,k,c)	
		Forecast value	APE(%)	Forecast value	APE(%)	Forecast value	APE(%)	Forecast value	APE(%)	Forecast value	APE(%)	Forecast value	APE(%)
*Training stage*													
2001	22,888.40	22,888.40	0.00	22,888.40	0.00	23,248.20	1.57	22,888.40	0.00	22,888.40	0.00	22,888.40	0.00
2002	24,789.20	26,735.05	7.85	24,900.39	0.45	25,420.52	2.55	24,789.20	0.00	24,789.20	0.00	24,791.69	0.01
2003	27,125.80	28,336.73	4.46	27,900.26	2.86	27,599.32	1.75	27,125.80	0.00	27,125.80	0.00	28,866.36	6.42
2004	31,700.50	30,034.36	5.26	30,431.76	4.00	29,784.60	6.04	31,700.50	0.00	31,700.50	0.00	31,344.86	1.12
2005	32,547.00	31,833.70	2.19	32,746.91	0.61	31,976.36	1.75	32,547.00	0.00	32,547.00	0.00	33,150.25	1.85
2006	34,876.20	33,740.84	3.26	34,950.86	0.21	34,174.59	2.01	33,704.23	3.36	35,051.04	0.50	34,857.97	0.05
2007	36,658.70	35,762.23	2.45	37,098.66	1.20	36,379.31	0.76	36,028.68	1.72	36,517.36	0.39	36,637.84	0.06
2008	37,302.90	37,904.73	1.61	39,223.32	5.15	38,590.50	3.45	38,608.23	3.50	37,376.01	0.20	38,564.13	3.38
2009	38,384.50	40,175.57	4.67	41,346.58	7.72	40,808.18	6.31	40,497.16	5.50	38,053.10	0.86	40,668.96	5.95
2010	44,101.00	42,582.47	3.44	43,483.85	1.40	43,032.33	2.42	41,798.82	5.22	38,300.44	13.15	42,962.50	2.58
2011	45,378.50	45,133.56	0.54	45,646.68	0.59	45,262.96	0.25	45,151.50	0.50	46,014.98	1.40	45,321.46	0.13
2012	47,797.30	47,837.48	0.08	47,844.17	0.10	47,500.07	0.62	48,048.54	0.53	47,981.56	0.39	47,782.52	0.03
2013	49,970.60	50,703.40	1.47	50,083.80	0.23	49,743.66	0.45	50,378.39	0.82	49,695.47	0.55	50,277.75	0.61
*Testing stage*													
2014	51,859.40	53,741.00	3.63	52,371.93	0.99	51,993.73	0.26	53,158.52	2.51	50,359.65	2.89	52,835.24	1.88
2015	55,960.20	56,960.59	1.79	54,714.16	2.23	54,250.27	3.06	55,903.91	0.10	52,804.11	5.64	55,371.19	1.05
2016	57,692.90	60,373.07	4.65	57,115.53	1.00	56,513.30	2.04	58,990.34	2.25	57,433.38	0.45	57,919.65	0.39
2017	60,395.90	63,989.98	5.95	59,580.69	1.35	58,782.80	2.67	61,919.58	2.52	59,137.13	2.08	60,444.52	0.08
2018	62,245.10	67,823.58	8.96	62,114.02	0.21	61,058.79	1.91	64,660.15	3.88	61,567.91	1.09	62,962.36	1.15

**Table 5:** Comparison of the prediction accuracies of the six models (2001–2018)

	MAPE(%)	NMAPE(%)	RMSE	NRMSE
*Training stage*				
GM(1,1)	2.87	2.64	3076.17	**0.03**
ONGBM(1,1)	1.89	1.86	1088.07	**0.03**
PR	2.30	2.19	1043.16	**0.03**
ANN	1.63	1.78	1019.75	**0.03**
SVM	**1.34**	**1.61**	1624.97	0.04
ONGBM(1,1,k,c)	1.71	1.65	**949.66**	**0.03**
*Testing stage*				
GM(1,1)	4.99	5.11	3339.50	0.06
ONGBM(1,1)	1.16	1.14	752.38	**0.01**
PR	1.99	2.02	1291.73	0.02
ANN	2.25	2.29	1518.44	0.03
SVM	2.43	2.38	1692.37	0.03
ONGBM(1,1,k,c)	**0.91**	**0.89**	**611.14**	**0.01**

The optimum values of the above metrics for two cases are in bold type

**Table 6:** Simulated and forecasted values of China’s petroleum terminal consumption using different models

Year	Data	GM(1,1)		ONGBM(1,1)		PR		ANN		SVR		ONGBM(1,1,k,c)	
		Forecast value	APE(%)	Forecast value	APE(%)	Forecast value	APE(%)	Forecast value	APE(%)	Forecast value	APE(%)	Forecast value	APE(%)
*Training stage*													
2001	22,888.40	22,888.40	0.00	22,888.40	0.00	23,248.20	1.57	22,888.40	0.00	22,888.40	0.00	22,888.40	0.00
2002	24,789.20	26,735.05	7.85	24,900.39	0.45	25,420.52	2.55	24,789.20	0.00	24,789.20	0.00	24,791.69	0.01
2003	27,125.80	28,336.73	4.46	27,900.26	2.86	27,599.32	1.75	27,125.80	0.00	27,125.80	0.00	28,866.36	6.42
2004	31,700.50	30,034.36	5.26	30,431.76	4.00	29,784.60	6.04	31,700.50	0.00	31,700.50	0.00	31,344.86	1.12
2005	32,547.00	31,833.70	2.19	32,746.91	0.61	31,976.36	1.75	32,547.00	0.00	32,547.00	0.00	33,150.25	1.85
2006	34,876.20	33,740.84	3.26	34,950.86	0.21	34,174.59	2.01	33,704.23	3.36	35,051.04	0.50	34,857.97	0.05
2007	36,658.70	35,762.23	2.45	37,098.66	1.20	36,379.31	0.76	36,028.68	1.72	36,517.36	0.39	36,637.84	0.06
2008	37,302.90	37,904.73	1.61	39,223.32	5.15	38,590.50	3.45	38,608.23	3.50	37,376.01	0.20	38,564.13	3.38
2009	38,384.50	40,175.57	4.67	41,346.58	7.72	40,808.18	6.31	40,497.16	5.50	38,053.10	0.86	40,668.96	5.95
2010	44,101.00	42,582.47	3.44	43,483.85	1.40	43,032.33	2.42	41,798.82	5.22	38,300.44	13.15	42,962.50	2.58
2011	45,378.50	45,133.56	0.54	45,646.68	0.59	45,262.96	0.25	45,151.50	0.50	46,014.98	1.40	45,321.46	0.13
2012	47,797.30	47,837.48	0.08	47,844.17	0.10	47,500.07	0.62	48,048.54	0.53	47,981.56	0.39	47,782.52	0.03
2013	49,970.60	50,703.40	1.47	50,083.80	0.23	49,743.66	0.45	50,378.39	0.82	49,695.47	0.55	50,277.75	0.61
*Testing stage*													
2014	51,859.40	53,741.00	3.63	52,371.93	0.99	51,993.73	0.26	53,158.52	2.51	50,359.65	2.89	52,835.24	1.88
2015	55,960.20	56,960.59	1.79	54,714.16	2.23	54,250.27	3.06	55,903.91	0.10	52,804.11	5.64	55,371.19	1.05
2016	57,692.90	60,373.07	4.65	57,115.53	1.00	56,513.30	2.04	58,990.34	2.25	57,433.38	0.45	57,919.65	0.39
2017	60,395.90	63,989.98	5.95	59,580.69	1.35	58,782.80	2.67	61,919.58	2.52	59,137.13	2.08	60,444.52	0.08
2018	62,245.10	67,823.58	8.96	62,114.02	0.21	61,058.79	1.91	64,660.15	3.88	61,567.91	1.09	62,962.36	1.15

**Table 7:** Comparison of the prediction accuracies of the six models in China’s petroleum terminal consumption

	MAPE(%)	NMAPE(%)	RMSE	NRMSE
*Training stage*				
GM(1,1)	2.84	2.10	1027.63	0.03
ONGBM(1,1)	2.36	1.76	1127.25	0.03
PR	2.12	1.58	826.00	0.02
ANN	2.75	2.08	1057.03	0.03
SVR	5.88	4.13	2227.24	0.06
ONGBM(1,1,k,c)	**1.58**	**1.18**	**851.53**	**0.02**
*Testing stage*				
GM(1,1)	7.93	7.71	4967.93	0.09
ONGBM(1,1)	3.01	2.91	1764.76	0.03
PR	7.64	7.46	5062.82	0.09
ANN	4.38	4.23	2561.14	0.05
SVR	2.51	2.33	2031.48	0.04
ONGBM(1,1,k,c)	**0.71**	**0.68**	**446.85**	**0.01**

The optimum values of the above metrics for two cases are in bold type

In Case 1, observed from Table 4 and Figs. 4 and 5, it is concluded that in the in-sample period, the intelligent methods (i.e., SVR and ANN) outperform other candidate models, because the modeling results generated by these two models are closer to the actual samples, followed by the grey models and polynomial regression. In addition, the range of APE of the intelligent methods is smaller than that of the grey-based models. While the performance of the mentioned competitive models will be changed when it comes to the out-of-sample period. The calculated results by the proposed model are closer to the actual values and its range of APE values is narrowest among these models.

Next, we compare four different error indicators (MAPE, NMAPE, RMSE, and NRMSE). The calculated errors are tabulated in Table 5. It is obviously seen from Table 5 that the MAPE and NMAPE of the SVR model are smallest, and RMSE and NRMSE of the ONGBM(1,1,k,c) model are lowest. On the contrary, the four error indicators of the newly proposed ONGBM(1,1,k,c) model are all lowest, and they are 0.91%, 0.89%, 611.14, and 0.01, respectively, in the test set. The newly designed model shows a higher accuracy with respect to forecasting China’s petroleum consumption.

In case 2, we conduct the two-step analysis similar to Case 1. It is seen from Table 6 and Fig. 6 that our calculated results are closer to the actual observations of China’s petroleum terminal consumption for both the training and test sets, and the newly designed model has a narrower changing range for APE values (Fig. 7), indicating that the performance stability of the proposed model is superior over other benchmarks.

Considering the four different error indices in Case 2, it is known from Table 7 that the newly designed ONGBM(1,1,k,c) model is more accurate, because its MAPE, NMAPE, RMSE, and NRMSE values are lowest for both the in-sample and out-of-sample periods. In particular, the competitors mentioned in this paper perform well in this case by reference with the Lewis standard [47]. Through the in-depth analysis, the GM(1,1) model and PR has comparatively poor forecasting abilities. And when GM(1,1) is further modified by Wang et al. [43], as a result, the forecasting result generated by ONGBM(1,1) can be improved. The intelligent methods can be treated as the alternative tools for forecasting Cases 1–2. Our results investigate that the newly designed ONGBM(1,1,k,c) model is the optimal model for forecasting China’s petroleum consumption in this case, and the reason for this situation is that the proposed model has a more flexible structure owing to its flexible fractional accumulated generation operator and power index.

### Out-of-sample predictions and suggestion

Our tests suggest that the newly designed ONGBM(1,1,k,c) model is more suitable for forecasting China’s petroleum consumption and terminal consumption than the competitive models. Therefore, the proposed model is selected as the optimal model for making out-of-sample predictions of China’s petroleum consumption and terminal consumption in the next 5 years, and the calculated results are listed in Table 8.

**Table 8:** Forecast results of China’s petroleum consumption and terminal consumption from 2019 to 2023 (10^4^ tons)

Year	Petroleum consumption	Terminal consumption
2019	64,704.31	60,820.74
2020	66,999.01	62,916.77
2021	69,279.32	64,993.39
2022	71,548.23	67,053.98
2023	73,808.16	69,101.31

According to the historical data and forecast data of petroleum consumption mentioned above, China’s petroleum consumption presents a trend of continuous growth. However, it can be seen from Fig. 2 that it is very difficult to carry out the exploration work of the petroleum in China, sustaining China’s petroleum exports at a very low level. Therefore, the supply–demand relationship of China’s petroleum will become more and more unbalanced. To avoid the contradiction between supply and demand affecting social development and national defense security, some suggestions and countermeasures for the development of China’s petroleum industry are gained as follows.

1. At present, the growth rate of China’s petroleum production is much lower than the growth rate of petroleum demand, and in recent years, petroleum production has shown a downward trend. This situation is unbelievable, because China has very large petroleum reserves. The problem that causes the unbalance of petroleum supply and demand mainly lies in the intensity of petroleum exploration. According to Fig. 2, the main reason for the difficulty in petroleum exploration is the uneven geographical distribution of China’s petroleum resources. Abundant petroleum resources are mostly stored in inaccessible places such as the Great Northwest and Northeast, where exploitation costs and difficulties are too great. Therefore, the petroleum industry should increase investment in petroleum exploitation and increase exploration and development efforts. At the same time, it is necessary to increase the input of logistics facilities and equipment, carry out crude petroleum pipeline transportation plans, and reduce the cost of crude petroleum transportation.
2. Figure 1 shows that the petroleum consumption of transportation, storage, and postal services has surpassed that of industry and has become the first industry in terms of petroleum consumption. Therefore, in the field of transportation, which belongs to the tertiary industry, the purchase of small-displacement cars can be encouraged; in the aspect of car design, energy-saving technology can be further studied to achieve the goal of energy-saving and emission reduction. In addition, related supporting facilities of new energy vehicles should be further improved, such as reasonable layout of charging piles, distribution stations, maintenance points, and other infrastructure, so as to solve the problem that cars cannot meet long-distance driving due to insufficient electric capacity. In addition, petroleum consumption in industry is second only to that of transportation, warehousing, and postal services. Therefore, China should adjust and upgrade its industrial structure, devote itself to developing high-tech and high value-added industries, and abandon the development of industries with high energy consumption.
3. By reference with the forecast results in the previous section, we can see that China’s overall petroleum consumption shows an increasing trend, while petroleum exploration will not play its due role immediately. Until China can quickly explore for petroleum, its main source of petroleum is still import. Therefore, China’s energy sector can reasonably import petroleum according to the forecast results of this paper, so as to avoid unnecessary waste caused by the excessive import of petroleum. In addition, the Chinese government can also sign agreements with major petroleum-exporting countries to continue imports, so that exporting countries can give certain discounts.

## Conclusion and research direction

To accurately predict China’s petroleum consumption, this paper proposes a newly optimized nonlinear grey Bernoulli model with combined fractional accumulated generation operator, and these varying parameters are optimized by marine predators algorithm for precisely excavating the development patterns of various time-series sequence. Specifically, we introduce a new combined conformable fractional accumulated generation operator into the existing modified nonlinear grey Bernoulli model. When we take the different values of the emerging coefficients (the fractional accumulation orders, background-value coefficient, and power index), the proposed model can be suitable for forecasting various time-series sequence issues.

As the numerical results in a series of cases show that the newly designed ONGBM(1,1,k,c) model does reach a higher accuracy in forecasting China’s coal consumption and petroleum consumption, compared with other competitive models that embrace the traditional grey model, optimized nonlinear grey Bernoulli model, polynomial regression, artificial neural network, and support vector regression. Our tests suggest that the newly proposed model should be considered the optimal technique for forecasting China’s petroleum consumption and terminal consumption in the next period. Based on the forecasts of this paper, some suggestions are recommended for the relevant sectors in formulating reasonable plans and strategies.

Up to this point, the newly optimized nonlinear grey Bernoulli model with combined conformable fractional accumulation has prominent advantages over the other benchmarks, whereas there remains room for improvement. For example, the proposed model is essentially a single variable-based model, neglecting relevant influential factor in practice. Additionally, other latest algorithms should be employed for improving the effectiveness of the proposed model in our next work.

## References

[1] Hensel ND (2012) An economic and national security perspective on critical resources in the energy sector. In: *New security frontiers: critical energy and the resource challenge*, p. 113–138.

[2] Wang Q, Su M, Li R (2018). Toward to economic growth without emission growth: the role of urbanization and industrialization in China and India. J Clean Prod.

[3] Zhang Z (2011). China’s energy security, the Malacca dilemma and responses. Energy Policy.

[4] Wang Q, Li S, Li R (2018). China’s dependency on foreign oil will exceed 80% by 2030: developing a novel NMGM-ARIMA to forecast China's foreign oil dependence from two dimensions. Energy.

[5] Turanoglu E, Senvar O, Kahraman C (2012). Oil consumption forecasting in Turkey using artificial neural network. Int J Energy Optim Eng (IJEOE).

[6] Dritsaki C, Niklis D, Stamatiou P (2021). Oil consumption forecasting using ARIMA models: an empirical study for Greece. Int J Energy Econ Policy.

[7] Yuan C, Zhu Y, Chen D, Liu S, Fang Z (2017). Using the GM (1, 1) model cluster to forecast global oil consumption. Grey Syst Theory Appl.

[8] Azadeh A, Khakestani M, Saberi M (2009). A flexible fuzzy regression algorithm for forecasting oil consumption estimation. Energy Policy.

[9] Alkhathlan K, Javid M (2015). Carbon emissions and oil consumption in Saudi Arabia. Renew Sustain Energy Rev.

[10] Al-Fattah SM, Aramco S (2021). Application of the artificial intelligence GANNATS model in forecasting crude oil demand for Saudi Arabia and China. J Petrol Sci Eng.

[11] Huang Y, Li S, Wang R, Zhao Z, Huang B, Wei B, Zhu G (2021). Forecasting oil demand with the development of comprehensive tourism. Chem Technol Fuels Oils.

[12] Yao T, Wang Z (2020). Crude oil price prediction based on LSTM network and GM (1, 1) model. Grey Syst Theory Appl.

[13] Lu SL, Tsai CF (2016). Petroleum demand forecasting for Taiwan using modified fuzzy-grey algorithms. Expert Syst.

[14] Yang Y, Chen Y, Shi J, Liu M, Li C, Li L (2016). An improved grey neural network forecasting method based on genetic algorithm for oil consumption of China. J Renew Sustain Energy.

[15] Hyndman RJ, Kostenko AV (2007). Minimum sample size requirements for seasonal forecasting models. Foresight.

[16] Ofosu-Adarkwa J, Xie N, Javed SA (2020). Forecasting CO_2_ emissions of China's cement industry using a hybrid Verhulst-GM (1, N) model and emissions' technical conversion. Renew Sustain Energy Rev.

[17] Liu C, Wu WZ, Xie W, Zhang J (2020). Application of a novel fractional grey prediction model with time power term to predict the electricity consumption of India and China. Chaos Solitons Fractals.

[18] Ding S, Li R, Wu S, Zhou W (2021). Application of a novel structure-adaptative grey model with adjustable time power item for nuclear energy consumption forecasting. Appl Energy.

[19] Zeng B, Ma X, Zhou M (2020). A new-structure grey Verhulst model for China’s tight gas production forecasting. Appl Soft Comput.

[20] Ma X, Mei X, Wu W, Wu X, Zeng B (2019). A novel fractional time delayed grey model with Grey Wolf Optimizer and its applications in forecasting the natural gas and coal consumption in Chongqing China. Energy.

[21] Liu C, Lao T, Wu W, Xie W, Zhu H (2022) An optimized nonlinear grey Bernoulli prediction model and its application in natural gas production. Expert Syst Appl 194: 116448.

[22] Javed SA, Zhu B, Liu S (2020). Forecast of biofuel production and consumption in top CO_2_ emitting countries using a novel grey model. J Clean Prod.

[23] Şahin U, Şahin T (2020). Forecasting the cumulative number of confirmed cases of COVID-19 in Italy, UK and USA using fractional nonlinear grey Bernoulli model. Chaos Solitons Fractals.

[24] Yoga I, Yudiarta IGA (2021). Grey forecasting of inbound tourism to bali and financial loses from the COVID-19. Int J Grey Syst.

[25] Arsy FA (2021). Demand forecasting of toyota avanza cars in indonesia: grey systems approach. Int J Grey Syst.

[26] Septyari FM (2021). Grey forecasting of the exports of indonesian palm oil to India. Int J Grey Syst.

[27] Cui J, Liu SF, Zeng B, Xie NM (2013). A novel grey forecasting model and its optimization. Appl Math Model.

[28] Ma X, Liu Z (2017). Application of a novel time-delayed polynomial grey model to predict the natural gas consumption in China. J Comput Appl Math.

[29] Qian WY, Dang YG, Liu SF (2012). Grey GM (1, 1, tα) model with time power and its application. Syst Eng Theory Pract.

[30] Wei B, Xie N, Hu A (2018). Optimal solution for novel grey polynomial prediction model. Appl Math Model.

[31] Liu C, Xie W, Wu WZ, Zhu H (2021). Predicting Chinese total retail sales of consumer goods by employing an extended discrete grey polynomial model. Eng Appl Artif Intell.

[32] Chen CI (2008). Application of the novel nonlinear grey Bernoulli model for forecasting unemployment rate. Chaos Solitons Fractals.

[33] Wu L, Liu S, Yao L, Yan S, Liu D (2013). Grey system model with the fractional order accumulation. Commun Nonlinear Sci Numer Simul.

[34] Xie W, Wu WZ, Liu C, Zhao J (2020). Forecasting annual electricity consumption in China by employing a conformable fractional grey model in opposite direction. Energy.

[35] Ma X, Wu W, Zeng B, Wang Y, Wu X (2020). The conformable fractional grey system model. ISA Trans.

[36] Şahin U (2021). Future of renewable energy consumption in France, Germany, Italy, Spain, Turkey and UK by 2030 using optimized fractional nonlinear grey Bernoulli model. Sustain Prod Consum.

[37] Liu C, Lao T, Wu WZ, Xie W (2021). Application of optimized fractional grey model-based variable background value to predict electricity consumption. Fractals.

[38] Wu W, Ma X, Zeng B, Lv W, Wang Y, Li W (2020). A novel Grey Bernoulli model for short-term natural gas consumption forecasting. Appl Math Model.

[39] Faramarzi A, Heidarinejad M, Mirjalili S, Gandomi AH (2020). Marine predators algorithm: a nature-inspired metaheuristic. Expert Syst Appl.

[40] Zeng B, Duan H, Zhou Y (2019). A new multivariable grey prediction model with structure compatibility. Appl Math Model.

[41] Candra CS, Adrian J, Lim VC (2021). Indonesian trade deficit with China: background and grey forecasting. Int J Grey Syst.

[42] Deng J (1982). System and control letter. Control Probl Grey Syst.

[43] Wang ZX, Hipel KW, Wang Q, He SW (2011). An optimized NGBM (1, 1) model for forecasting the qualified discharge rate of industrial wastewater in China. Appl Math Model.

[44] Ostertagová E (2012). Modelling using polynomial regression. Proc Eng.

[45] Sinha SK, Wang MC (2008). Artificial neural network prediction models for soil compaction and permeability. Geotech Geol Eng.

[46] Smola AJ, Schölkopf B (2004). A tutorial on support vector regression. Stat Comput.

[47] Lewis CD (1982). Industrial and business forecasting methods: a practical guide to exponential smoothing and curve fitting.

